# Hu-lu-su-pian ameliorates hepatic steatosis by regulating CIDEA expression in AKT-driven MASLD mice

**DOI:** 10.3389/fphar.2024.1503247

**Published:** 2025-01-31

**Authors:** Rumeng Ren, Qi Wang, Dongjie Deng, Aoao Guo, Xin Chen, Yan Meng, Ying Fang, Guohua Zheng, Zhong Xu, Man Li, Junjie Hu

**Affiliations:** ^1^ School of Pharmacy, Hubei University of Chinese Medicine, Wuhan, Hubei, China; ^2^ Hubei Shizhen Laboratory, Wuhan, Hubei, China; ^3^ Department of Gastroenterology, Zhongnan Hospital of Wuhan University, Health Management Center, Zhongnan Hospital of Wuhan University, Wuhan, Hubei, China; ^4^ Guizhou Provincial People’s Hospital, Guiyang, Guizhou, China; ^5^ Department of Integrated Traditional and Western Medicine, Hubei Cancer Hospital, Tongji Medical College, Huazhong University of Science and Technology, Wuhan, Hubei, China

**Keywords:** Hu-lu-su-pian, metabolic dysfunction-associated steatotic liver disease, CIDEA, de novo fatty acid synthesis, lipid droplets

## Abstract

**Introduction:**

Hu-lu-su-pian (HLSP) is an oral tablet derived from the active compounds of *Cucumis melo* L., a traditional Chinese medicine. This contemporary formulation is frequently employed in clinical settings for the management of liver ailments. However, the molecular mechanism by which HLSP affects metabolic dysfunction-associated steatotic liver disease (MASLD) remains unclear. This study aimed to explore the therapeutic potential of HLSP on MASLD and the underlying mechanism.

**Methods:**

The researchers used ultra-high-performance liquid chromatography coupled with quadrupole time-of-flight tandem mass spectrometry (UPLC-Q-TOF-MS/MS) to identify the primary chemical components of HLSP. A mouse model of MASLD induced by AKT was established through hydrodynamic transfection with activated forms of AKT. Serum biochemical indices and liver pathological assessments were employed to evaluate the pharmacodynamic effects of HLSP on MASLD. Transcriptomic analysis of the liver was conducted to detect differentially expressed genes (DEGs). Further examination of significant DEGs and proteins was performed using quantitative real-time polymerase chain reaction (RT-qPCR), Western blotting, and immunohistochemistry (IHC) techniques, respectively. The efficacy and molecular mechanisms of HLSP in MASLD were further explored in HepG2 and Huh-7 cells in the presence of gene overexpression.

**Results:**

From the UPLC-Q-TOF-MS/MS results, we detected fifteen components from HLSP. From the results of serum biochemical indices and hepatic pathology analyses, it is clear that HLSP is effective in treating MASLD. The findings from hepatic transcription studies revealed CIDEA as an essential DEG that facilitates lipid droplet (LD) fusion and enhances *de novo* fatty acid synthesis from scratch in cases of hepatic steatosis, which HLSP has the potential to counteract. In addition, HLSP significantly reduced lipid accumulation and expression of critical genes for *de novo* fatty acid synthesis in HepG2 and Huh-7 cells overexpressing CIDEA.

**Discussion:**

The present study preliminarily suggests that HLSP can ameliorate hepatic steatosis by inhibiting CIDEA-mediated *de novo* fatty acid synthesis and LD formation, which may offer a potential strategy for treating MASLD.

## 1 Introduction

Metabolic dysfunction-associated steatotic liver disease (MASLD) stands out as the most prevalent liver disorder, identified by the presence of hepatocellular steatosis and occurring in individuals with no significant history of alcohol use (Rinella, 2015). MASLD is a condition that can be reversed; however, if it goes untreated, it may advance to metabolic dysfunction-associated steatohepatitis (MASH) and subsequent liver fibrosis (Ratziu and Poynard, 2006). As a common chronic liver disease worldwide, MASLD represents a considerable economic challenge for the global population. A recent study found that the worldwide prevalence of MASLD stands at about 32.4% and continues to rise (Riazi et al., 2022). Consequently, investigating how drugs function and their impact in both preventing and treating MASLD is of utmost importance.

Fatty liver or steatosis is a condition in which lipids accumulate excessively in the liver and is a hallmark of MASLD (Vergani, 2019). Hepatic lipids can originate from dietary sources, plasma free fatty acids (FFA) esterification, or hepatic *de novo* fatty acid synthesis (Softic et al., 2016). In the *de novo* fatty acid synthesis, the process begins with acetyl-coenzyme A, which is transformed by crucial enzymes like acetyl-CoA carboxylase (ACC) and fatty acid synthase (FASN) to generate lipids, the primary form of energy reserve (Batchuluun et al., 2022). ACC and FASN, key enzymes in fatty acid synthesis, control the level of fatty acid production (Guo et al., 2024). Under conditions such as obesity, *de novo* fatty acid synthesis experiences dysregulation and a marked increase, leading to excessive triglyceride (TG) accumulation or hepatic steatosis. This represents an early stage in the development of MASLD and MASH (Fabbrini et al., 2010; Friedman et al., 2018; Smith et al., 2020; Tilg et al., 2021). Lambert et al. reported a 26% contribution of *de novo* fatty acid synthesis to TG in adults with MASLD, a threefold increase in this contribution compared with adults without MASLD (Lambert et al., 2014). A growing collection of research indicates that heightened *de novo* fatty acid synthesis in liver cells plays a vital role in the advancement of MASLD. Consequently, the strategic reduction of hepatic lipogenesis has been acknowledged for some time as an effective therapeutic approach for managing MASLD and MASH (Musso et al., 2016; Rinella and Sanyal, 2016; Neuschwander-Tetri, 2020).

Lipid droplets (LDs) are round organelles that are commonly located in the cytoplasm across diverse cell types. They primarily function as reservoirs for TG and cholesteryl esters, characterized by a hydrophobic core of neutral lipids surrounded by a single layer of phospholipids embellished with a distinct collection of proteins. These droplets play an essential role in regulating lipid homeostasis within the body and are associated with the development of numerous health conditions (Chen et al., 2020). MASLD is distinguished by an overabundance of TG within LDs (Tsai et al., 2017), rendering LDs a hallmark characteristic of hepatic steatosis (Schott et al., 2019). Disorders related to lipid metabolism contribute to the onset of fatty liver by leading to an overaccumulation of fatty acids stored as LDs within liver cells. This buildup of LDs intensifies oxidative stress and inflammation in the liver, which subsequently causes ongoing damage and paves the way for more severe conditions, such as MASH (Chen et al., 2019). Thus, regulating LD production is crucial for preventing and treating MASLD.

Cell death-inducing DNA break factor-alpha-like effectors (CIDEs), which include proteins like CIDEA, CIDEB, and CIDEC/Fsp27, represent a specialized group that is intricately linked to lipid metabolism. These proteins are essential for the functionality of adipocytes and hepatocytes, especially at LD-LD contact sites, where they promote the selective transfer of lipids from smaller to larger droplets (Gao et al., 2017). They play a crucial role in controlling the merging and development of unusual LDs, acting as essential regulators in the creation of sizable LDs within adipocytes and hepatocytes (Gong et al., 2011; Xu et al., 2016).

The expansion of LDs leads to larger LDs, thereby diminishing the surface area-to-volume ratio of the aggregate LDs population. This decline in the ratio impedes lipolysis, as it diminishes the availability of lipase molecules for attachment to the LDs surface. Consequently, the growth of LDs enhances the capacity for lipid storage and fosters the accumulation of intracellular lipids (Chen et al., 2020). However, excessive lipid uptake can lead to uncontrolled growth of LDs (hypertrophy) in fatty tissues or the liver, potentially causing metabolic diseases like obesity and fatty liver disease (Krahmer et al., 2013; Rosen and Spiegelman, 2014; Gluchowski et al., 2017). CIDEA is found to be prominently expressed in steatotic livers in both humans and mice. Zhou and colleagues discovered that when CIDEA is overexpressed in the AML12 hepatocyte cell line, there is a buildup of larger LDs (Zhou et al., 2012). They also noted that CIDEA gene-deficient mice on a high-fat diet exhibited decreased levels of FASN and ACC in their livers. In addition, the heightened expression of CIDEA led to an upsurge in FASN and ACC levels across several non-hepatic cell types, while the reduction of CIDEA markedly suppressed the expression of both FASN and ACC (Sun et al., 2019; Cheng et al., 2022).

Hu-lu-su-pian (HLSP) is an oral tablet made from cucurbitacins, the active compounds of the traditional Chinese medicine, *Cucumis melo* L. This contemporary formulation is frequently employed in clinical settings to manage liver ailments. Cucurbitacins are recognized as key components in traditional Chinese medicine, boasting a diverse array of pharmacological benefits, including anti-inflammatory, antioxidant, hepatoprotective, and antitumor properties (Bartalis and Halaweish, 2011; Varela et al., 2022; Zhang et al., 2022; Dai et al., 2024). Plants that contain cucurbitacin, like *C. melo* and *Melonispedicellus*, have long been utilized in traditional medicine to address jaundice and liver cirrhosis, thanks to their protective and healing effects on the liver (Bartalis and Halaweish, 2011). In 1998, Cucurbitacin tablets (HLSP, approval number: Z12020128) were approved by the China Food and Drug Administration for the adjuvant treatment of chronic hepatitis and primary hepatocellular carcinoma (Wang et al., 2017; Liu et al., 2024), with the efficacy of inducing diuresis and removing yellowing, detoxifying and clearing heat. Cucurbitacin B and cucurbitacin E are the main active ingredients of HLSP (Ding et al., 2015). Numerous studies have demonstrated that cucurbitacin B and cucurbitacin E possess hepatoprotective effects (Wu et al., 2016; Sallam et al., 2018; Li et al., 2024). Nevertheless, the precise molecular mechanism of HLSP alleviates hepatic steatosis in rodent studies continues to elude straightforward elucidation.

This research employed UPLC-Q-TOF-MS/MS for a qualitative analysis of the primary constituents in HLSP and developed a mouse model of hepatic steatosis to explore the pharmacological effects of HLSP. The model exhibited a significant rise in liver lipogenesis due to the transfection of an activated variant of AKT, leading to liver-targeted overexpression of AKT. Subsequently, hepatic transcriptomics revealed the pharmacodynamic mechanism, and the role of the pharmacodynamic substances was further investigated at the cellular level.

## 2 Materials and methods

### 2.1 Chemicals and reagents

HLSP was provided by Tianjin Institute of Pharmaceutical Research Co., Ltd. (Tianjin, China). Protease inhibitors and phosphatase inhibitors were purchased from Jiangsu Cowi n Biotech Co., Ltd. (Jiangsu, China). The Cell Counting Kit-8 (CCK-8) was supplied by Dojindo Laboratories (Kyushu, Japan). ECL ultrasensitive chemiluminescent solution was acquired from Shanghai Epizyme Biomedical Technology Co., Ltd. (Shanghai, China). The CIDEA plasmid was produced by OriGene (Rockville, MD, USA) and its sequence can be accessed at https://www.origene.com.cn/drawmapbysku?SKU=RC205790.

### 2.2 Animals and treatments

Wild-type (WT) female FVB/N mice, between the ages of 6 and 8 weeks, were obtained from Charles River Laboratories located (Beijing, China). Before formal experiments, mice were properly fed for 7 days to acclimatize to the feeding conditions. Twenty-eight mice were randomly divided into 4 groups (n = 7 in each group): (1) WT group; (2) AKT group; (3) AKT-HLSP-L group (HLSP, 1.2 mg/kg/day); (4) AKT-HLSP-H (HLSP, 2.4 mg/kg/day). Hydrodynamic injections were performed on the latter 3 groups. The procedure was as follows: pT3-EF1α-HA-myr-AKT and pCMV/Sleeping Beauty transposase (SB) vectors were dissolved in 2 mL of 0.9% saline at a ratio of 25:1, and then filtered through a 0.22 μm micropore membrane, and then injected into the body through the tail vein of the mice in a short period of time (5–9 s). The pT3-EF1α-HA-myr-AKT plasmid (Addgene plasmid # 31789; http://n2t.net/addgene:31789; RRID:Addgene_31789) and the pCMV/SB vector (the SB gene information of which contains the sequence from 1863 to 2,881 in the CDS region of GenBank: MT559074.1) were generously donated by Dr. Xin Chen from the UCSF School of Pharmacy. HLSP (0.5% CMC-Na as the administration solvent, 1.2 or 2.4 mg/kg/day) was given by gavage to the administered group of mice within 5 weeks after plasmid injection. At the same time, mice in the WT and AKT groups were given equal amounts of 0.5% CMC-Na.

### 2.3 UPLC-Q-TOF-MS/MS identification

HLSP was ground, methanol was added, and then sonicated for 30 min. The resultant filtrate was employed for subsequent analytical procedures. We utilized an Agilent Zorbax SB-C18 RRHD column, measuring 2.1 × 100 mm with a fine particle size of 1.8 μm. The separation took place at a cozy 35°C, maintaining a steady flow rate of 0.2 mL/min. For the mobile phases, we employed a mix of water infused with 0.1% formic acid (referred to as solvent A) and acetonitrile (known as solvent B). The gradient elution procedure danced through time as follows: 0–6 min, 15%–45% B; 6–16 min, 45%B; 16–20 min, 45%–85% B; 20–28 min, 85% B; 28–29 min, 85%–15% B; 29–36 min, 15% B. The injected volume was 5 μL. An electrospray ionization source (ESI) was used to acquire data for HLSP in positive ion mode.

### 2.4 Biochemical analysis

In accordance with the protocols outlined in the accompanying test kits, we measured the concentrations of serum total cholesterol (TC), triglyceride (TG), alanine aminotransferase (ALT), and aspartate aminotransferase (AST).

### 2.5 Histopathological examination

Utilizing a microtome, we prepared sections of liver tissue that had been embedded in paraffin, subsequently staining them with hematoxylin-eosin (H&E). For the ORO staining process, we employed a cryostat to obtain frozen liver sections, which we then dyed with ORO. These samples were mounted in glycerol gelatin and examined microscopically, with images captured for later analysis.

### 2.6 Immunohistochemistry (IHC)

Liver tissues that had been embedded in paraffin were sliced into 4 µm sections, placed in an oven at 60 °C overnight to facilitate baking, then deparaffinized using xylene, and dehydrated through a gradient of alcohol. After rinsing with PBS, the tissues underwent antigen retrieval using citrate buffer. Then, the sections were blocked with 3% H_2_O_2_ to block endogenous peroxidase. The sections were incubated for 30 min at room temperature with a drop of 10% goat serum sealing solution. After adding the primary antibody, the sections were placed at 4 °C overnight. On a subsequent day, the secondary antibody was added and incubated, then washed with PBS, and then color developer was added to develop the color. The immunohistochemical sections were observed and photographed by an inverted light microscope.

### 2.7 Western blotting

Total protein was obtained by treating tissues or cells with appropriate amounts of RIPA lysate (containing protease inhibitors and phosphatase inhibitors). Protein concentrations were obtained using the BCA Protein Assay Kit. After the protein samples were prepared, an equal mass of protein was loaded onto sodium dodecyl sulphate-polyacrylamide gels (SDS-PAGE) (20–50 μg/lane), and the concentrated gels were electrophoresed at 60 V for 30 min, and then the voltage was changed to 100 V when the protein samples were in the separating gels until the proteins were completely separated. The gel was transferred to PVDF membrane, and the transfer voltage was set at 25 V and the transfer time was 30 min (the transfer time varied according to the molecular weight of the protein). After membrane transfer, 5% skimmed milk was blocked at room temperature for 1 h. The washed PVDF membrane was incubated in diluted primary antibodies (CIDEA, Proteintech, 13170-1-AP; FASN, CST, 3,180; ACC, CST, 3,676; and β-actin, CST, 4,970) in a shaker at 4 °C overnight. The membrane was washed with TBST and incubated with HRP-labelled secondary antibody in a horizontal shaker at room temperature for 1 h. The unbound secondary antibody was washed with PBST and developed with ECL luminescent solution in a dark room, and the protein immunoblot was obtained by using the GBOX Imaging Analysis System. All primary antibodies mentioned in this article are shown in Supplementary Table S1.

### 2.8 Real-time quantitative polymerase chain reaction (RT–qPCR) analysis

Total RNA was extracted from tissues and cells using TRIzol reagent. For every 1 mL of TRIzol, 0.2 mL of chloroform was added, and the mixture was incubated at room temperature for 2–3 min. After centrifugation, the upper aqueous layer was transferred to a clean centrifuge tube. An equal volume of isopropanol was added, followed by centrifugation, and the supernatant was discarded. The pellet was washed with 75% ethanol. The tube was then left at room temperature to allow the ethanol to evaporate, thus obtaining the RNA. Reverse transcription products were prepared according to the kit instructions and RT-qPCR detection was carried out. The gene amplification conditions were as follows: pre-denaturation at 95 °C for 2 min; denaturation at 95 °C for 10 s; annealing at 62 °C for 30 s; extension at 72 °C for 15 s, with 40 cycles repeated. The primer pairs used are shown in Supplementary Tables S2.

### 2.9 Cell culture and treatment

Human hepatocellular carcinoma cell lines (HepG2 and Huh-7) were used with *in vitro* experiments. Cell cultures were established using DMEM supplemented with 10% FBS and a mixture of antibiotics. The *in vitro* cellular concentrations of HLSP were meticulously scrutinized utilizing the CCK-8 assay to facilitate the selection of optimal conditions. Using the screened concentration of HLSP administration, the cells were inoculated in 60 mm dishes, and the EV group (pCMV6-Entry Vector), Over-CIDEA group, Over-CIDEA-HLSP-L group, and Over-CIDEA-HLSP-H group, were set up. To model CIDEA overexpressing cells, cells were cultured to 60% confluency. Then, 10 μg of CIDEA plasmid or pCMV6-Entry Vector was diluted with 1 mL of Opti-MEM, and 30 mL of Mega Tran 2.0 was added to the solution. The solution was gently vortexed to mix the solution and incubate at room temperature for 10–15 min to form the transfection complex. Add the transfection complex dropwise onto the cells and shake gently. Following a 12 h incubation period, the cells were supplied with new DMEM medium enriched with 10% FBS and were allowed to incubate for an additional 12 h. Subsequently, the cells were subjected to the judiciously selected HLSP concentration for 24 h. The HLSP treatment process involved the following steps: HLSP powder was weighed and dissolved in methanol, followed by sonication for 30 min. The resulting solution was filtered and evaporated until dry. After that, a specific amount of the dried HLSP was precisely weighed and dissolved in DMSO to prepare for subsequent cellular experiments.

### 2.10 BODIPY 493/503 staining

HepG2 and Huh-7 cells were inoculated in 6-well plates and allowed to proliferate to a density of approximately 60%. Subsequently, the cells were treated with HLSP for 24 h, as described previously. The cells were first fixed to preserve their structure, followed by staining with BODIPY 493/503 to visualize LDs. Subsequently, CIDEA was detected using specific primary antibodies, which were then recognized by fluorescent secondary antibodies sourced from Molecular Probes. Cells were observed under a fluorescence microscope.

### 2.11 ORO staning of HepG2 and Huh-7 cells

Upon achieving a cell density of approximately 60%, the cells underwent the treatment protocol previously outlined. Subsequently, they were stained by the guidelines provided by the ORO Staining Kit, specifically designed for cellular applications. The stained cells were then meticulously examined under a microscope and documented through photographic means.

### 2.12 Transcriptomics

The livers of different groups of mice were collected and their total RNA was extracted separately. Tests were performed to determine the quality of the RNA obtained. Metavir Biotech (Wuhan, China) used the Illumina sequencing platform to sequence complementary DNA (cDNA) libraries. We filtered the raw data using fastp v 0.19.3. Gene alignment and FPKM were calculated using feature Counts v1.6.2. We conducted a follow-up analysis of between-group differences based on previous studies (Du et al., 2024). In addition, we performed KEGG and GO analyses. Finally, we analyzed protein-protein interactions (PPIs) of DEGs.

### 2.13 Statistical analysis

The data collected in this study underwent statistical analysis through GraphPad Prism version 9.0, utilizing one-way ANOVA and t-tests for evaluation. *p* < 0.05 was considered statistically different.

## 3 Results

### 3.1 Qualitative analysis of main components of HLSP

**FIGURE 1:**
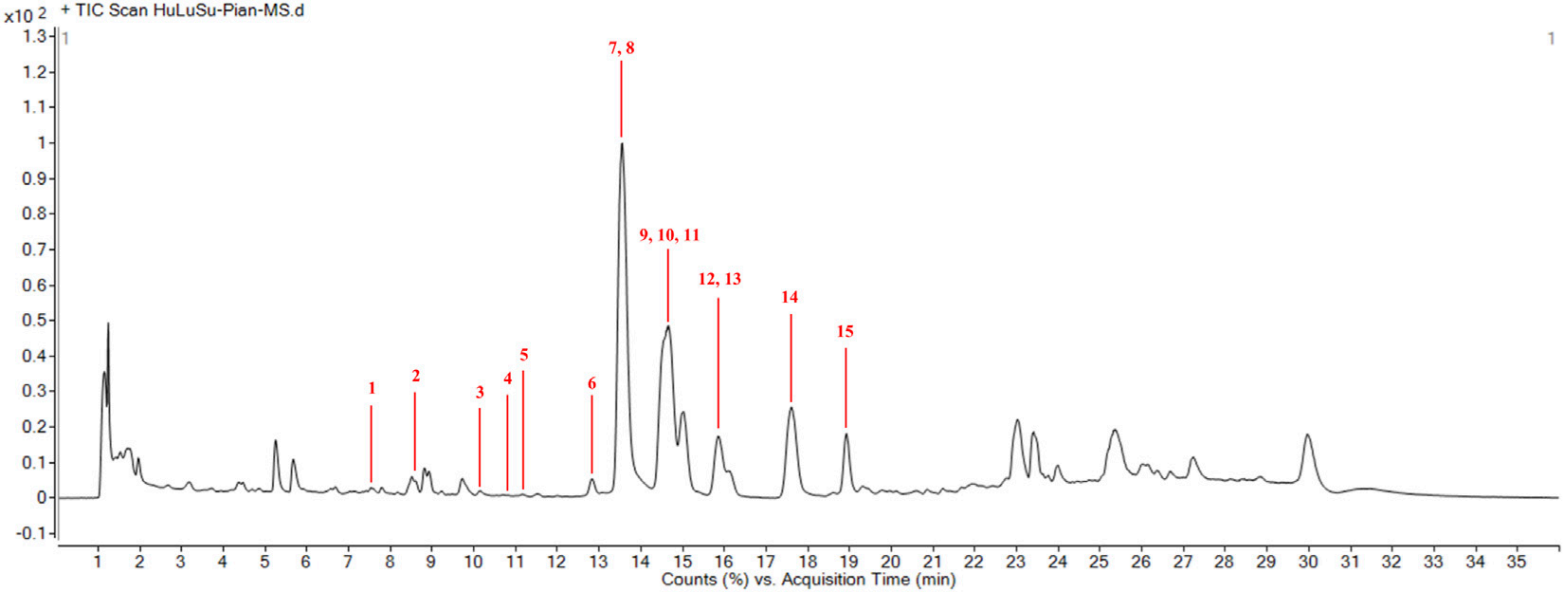
Qualitative analysis of main compnents of HLSP. (1) cucurbitacin D; (2) 23,24-dihydrocucurbitacin D; (3) 23,24-dihydroisocucurbitacin D; (4) isocucurbitacin D; (5) cucurbitacin I; (6) 3-epi-isocucurbitacin D; (7) cucurbitacin S; (8) cucurbitacin B; (9) 23,24-dihydrocucurbitacin B; (10) deacetylcucurbitacin B; (11) isocucurbitacin B; (12) 23,24-dihydroisocucurbitacin B; (13) deacetylisocucurbitacin B; (14) cucurbitacin E and (15) 3-epi-isocucurbitacin B.

Preliminary screening of the major components of HLSP was performed by UPLC-Q-TOF-MS/MS. Figure 1 illustrates the overall ion flow in positive ion mode, and fifteen active components were identified, namely, cucurbitacin D, 23,24-dihydrocucurbitacin D, 23,24-dihydroisocucurbitacin D, isocucurbitacin D, cucurbitacin I, 3-epi-isocucurbitacin D, cucurbitacin S, cucurbitacin B, 23,24-dihydrocucurbitacin B, deacetylcucurbitacin B, isocucurbitacin B, 23,24-dihydroisocucurbitacin B, deacetylisocucurbitacin B, cucurbitacin E and 3-epi-isocucurbitacin B. Table 1 shows the fragmentation information of each component. The structural formulas and secondary fragment ion diagrams of the components are comprehensively presented in Supplementary Figure S1.

**TABLE 1:** Fragmentation information for the main components of HLSP.

No.	RT (time)	Model	Compounds	Molecular formulae	Measured value (m/z)	Molecular weight (m/z)	Mass error (ppm)	MS/MS (m/z)
1	7.549	[M + H] +	Cucurbitacin D	C_30_H_44_O_7_	517.3160	517.3160	0.00	499.3025, 481.2938, 463.2846
2	8.855	[M + Na] +	23,24-dihydrocucurbitacin D	C_30_H_46_O_7_	541.3135	541.3091	8.13	541.3157
3	10.392	[M + Na] +	23,24-dihydroisocucurbitacin D	C_30_H_46_O_7_	541.3135	541.3135	0.00	523.3032
4	10.851	[M + H] +	Isocucurbitacin D	C_30_H_44_O_7_	517.3160	517.3131	5.61	517.3109, 499.3055
5	11.304	[M + H] +	Cucurbitacin I	C_30_H_42_O_7_	515.3003	515.2988	2.01	515.3042, 497.2869, 479.2760
6	12.865	[M + H] +	3-epi-isocucurbitacin D	C_30_H_44_O_7_	517.3160	517.3148	2.32	517.3160, 499.3039, 481.2925
7	13.462	[M + H] +	Cucurbitacin S	C_30_H_42_O_6_	499.3054	499.3061	−1.40	499.3063, 481.2948, 463.2850, 445.2736
8	13.672	[M + Na] +	Cucurbitacin B	C_32_H_46_O_8_	581.3084	581.3082	0.34	581.3050, 521.2818
9	14.439	[M + Na] +	23,24-dihydrocucurbitacin B	C_32_H_48_O_8_	583.3241	583.3235	1.03	583.3241, 523.3019
10	14.532	[M + H] +	Deacetylcucurbitacin B	C_30_H_44_O_6_	501.3211	501.3211	0.00	501.3215, 483.3110, 465.3015
11	14.651	[M + Na] +	Isocucurbitacin B	C_32_H_46_O_8_	581.3084	581.3084	0.00	581.3060,5 21.2780
12	15.746	[M + Na] +	23,24-dihydroisocucurbitacin B	C_32_H_48_O_8_	583.3241	583.3232	1.54	583.3248, 523.2985
13	15.802	[M + H] +	Deacetylisocucurbitacin B	C_30_H_44_O_6_	501.3211	501.3210	0.20	501.3214, 483.3100, 465.2990
14	17.539	[M + Na] +	Cucurbitacin E	C_32_H_44_O_8_	579.2928	579.2911	2.93	579.2896, 519.2615
15	18.860	[M + Na] +	3-epi-isocucurbitacin B	C_32_H_46_O_8_	581.3084	581.3076	1.38	581.3062, 521.2766

### 3.2 HLSP ameliorates AKT-driven hepatic steatosis in mice

**FIGURE 2:**
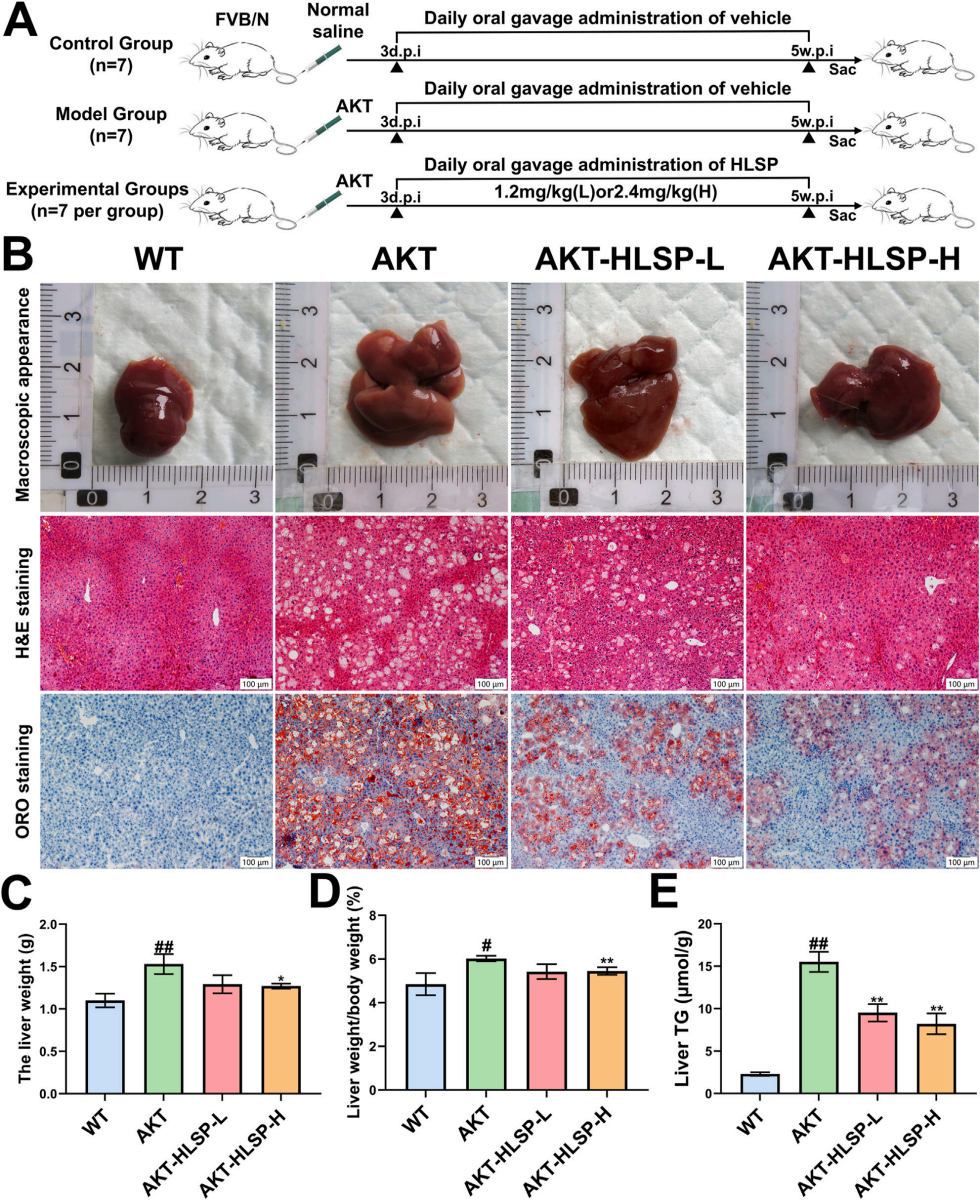
HLSP ameliorates AKT-driven hepatic steatosis in mice. **(A)** Study design; **(B–E)** macroscopic appearance **(B)**, H&E, and ORO staining of liver tissues; liver weight **(C)**; liver weight/body weight ratios **(D)**; and liver TG levels **(E)** from the wild-type (WT) or AKT-driven mice with intragastric administration of either vehicle or HLSP, respectively (Scale bar: 100 μm). Mean ± SD and ^#^ *P* < 0.05 versus the WT group and *P < 0.05 and **P < 0.01 versus the AKT group. Abbreviations: d.p.i., days postinjection; w.p.i., weeks post-injection; and TG, triglyceride.

Research has indicated that an increased expression of AKT in the liver triggers abnormal *de novo* fatty acid production, which can ultimately result in significant hepatic steatosis (Qiu et al., 2019). To elucidate the efficacy of HLSP, HLSP or vector was administered by gavage to mice 5 weeks after plasmid injection (Figure 2A). Mice injected with AKT displayed signs of fatty liver and an increase in liver size (Figure 2B). Both the absolute liver weight and liver/body weight ratio were elevated in comparison to the WT group (Figures 2C, D). During the 5 weeks of HLSP administration, HLSP showed a favorable amelioration of hepatic lipid accumulation compared to the AKT group. H&E and ORO staining showed massive balloon-like degeneration of hepatocytes and generation of a large number of LDs in the AKT group, whereas the HLSP group showed a significant degree of improvement in both (Figure 2B). Similarly, liver weight and liver/body weight ratio were lower in the HLSP group compared to the AKT group (Figures 2C, D), and intrahepatic TG levels were also significantly decreased (Figure 2E). These results suggested that HLSP slowed down hepatic steatosis in AKT-driven mice by reducing hepatic lipid accumulation.

### 3.3 HLSP improves liver function in AKT-driven hepatic steatosis mice

**FIGURE 3:**
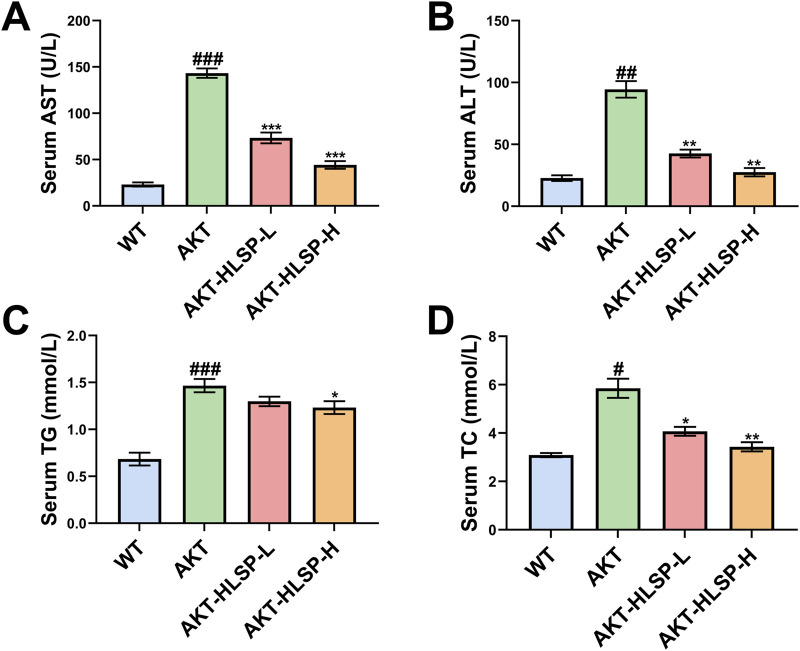
HLSP improves liver function in AKT-driven hepatic steatosis mice. **(A)** Serum AST and **(B)** ALT and **(C)** triglyceride and **(D)** total cholesterol levels from the wild-type (WT) or AKT-driven mice with oral administration of either vehicle or HLSP, respectively. Mean ± SD; ^#^ *P* < 0.05, ^##^ *P* < 0.01, and ^###^ *P* < 0.001 versus the WT group and *P < 0.05, **P < 0.01, and ***P < 0.001 versus the AKT group.

ALT and AST are surrogate markers for MASLD (Haigh et al., 2022). We found that both serum ALT and AST levels were significantly elevated in mice after AKT injection compared to the WT group. In contrast, after 5 weeks of HLSP administration, both serum ALT and AST decreased significantly (Figures 3A, B). In addition, we detected that HLSP treatment reduced serum TG and TC levels in AKT mice (Figures 3C, D). The above data suggest that HLSP improves liver function and lipid metabolism in AKT-driven mice.

### 3.4 HLSP is involved in the regulation of genes associated with lipid metabolism

**FIGURE 4:**
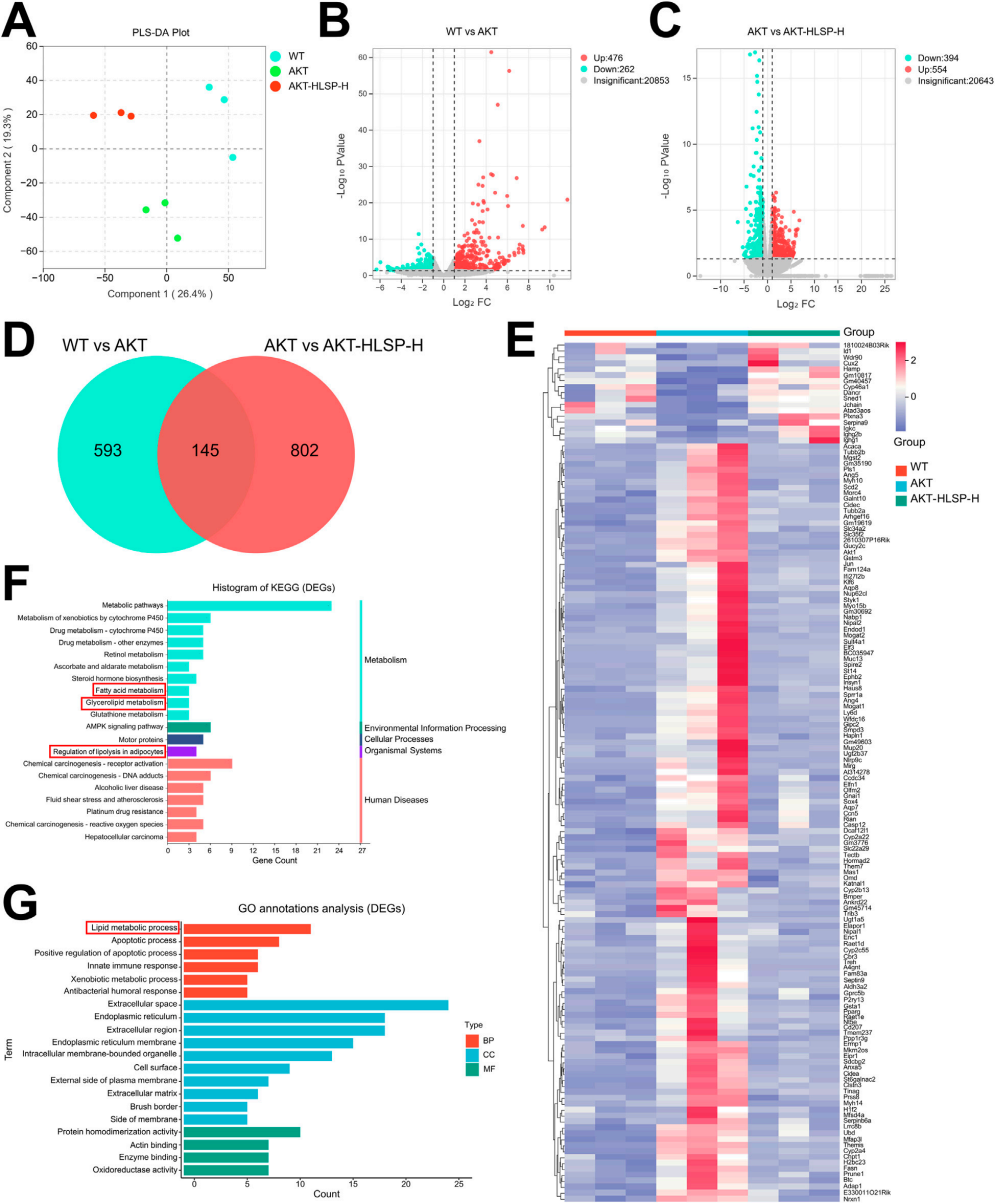
Hepatic transcriptomics analysis. **(A)** PCA of each group (n = 3). **(B)** Volcano plot of DEGs in the WT and AKT groups. **(C)** Volcano plot of DEGs in the AKT and AKT-HLSP-H groups. **(D)** Veen diagram of common DEGs in the WT, AKT, and AKT-HLSP-H groups. **(E)** Cluster heatmap of common DEGs in the three groups. **(F, G)** KEGG and GO annotations of common DEGs. Differentially expressed genes, DEGs; Principal-component analysis, PCA; Kyoto Encyclopedia of Genes and Genomes, KEGG; Gene Ontology, GO.

To further explore the potential mechanism of action of HLSP in ameliorating hepatic steatosis in mice, we next performed hepatic transcriptomic analysis. Principal component analysis (PCA) identified unique transcriptomic profiles among the three groups without any outliers (Figure 4A). The volcano plot additionally highlighted the differences in gene expression across the groups, showing 476 genes upregulated and 262 genes downregulated between the WT and AKT groups (Figure 4B). Similarly, 554 upregulated genes and 394 downregulated genes were present in the AKT group versus the AKT-HLSP-H group (Figure 4C). We established two gene clusters: one grouping WT with AKT and the other grouping AKT with AKT-HLSP-H. These clusters facilitated the identification of common genes that exhibited differential expression across all three groups. The Veen diagram (Figure 4D) shows the intersecting differential genes of the three groups, i.e., the potential genes for HLSP to ameliorate hepatic steatosis in mice. The clustering heatmap revealed that, compared to the AKT group, 17 DEGs were downregulated and 128 DEGs were upregulated in both the WT and AKT-HLSP-H groups (Figure 4E). We then performed KEGG and GO annotations. The KEGG annotation analysis indicated that DEGs primarily contribute to lipid metabolism. This encompasses the regulation of lipolysis in adipocytes, fatty acid metabolism and glycerolipid metabolism (Figure 4F). Similarly, GO annotation analyses revealed that the function of DEGs in terms of the biological processes was mainly focused on lipid metabolism (Figure 4G).

### 3.5 CIDEA is a key DEG for HLSP to improve hepatic steatosis in mice

**FIGURE 5:**
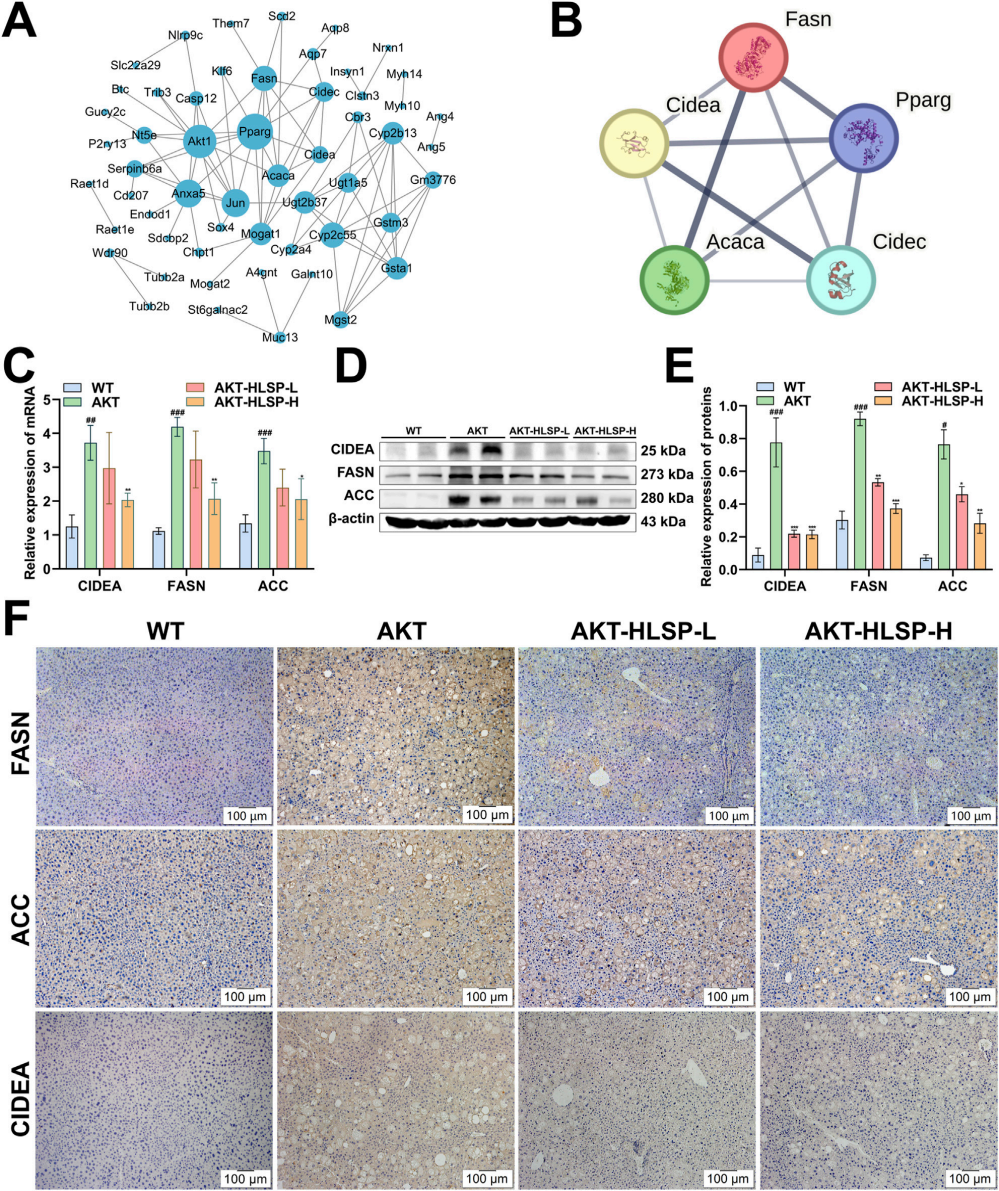
HLSP attenuates hepatic steatosis in AKT-driven mice by regulating the expression of CIDEA, FASN and ACC. **(A)** PPI network plot of key DEGs, medium confidence (0.4). **(B)** STRING network of key DEGs related to lipid metabolism, medium confidence (0.4). **(C)** mRNA expressions of CIDEA, FASN, and ACC in mouse livers were measured by RT-qPCR. **(D, E)** Comparison of active CIDEA, FASN, and ACC levels in mouse livers shown in representative immunoblots **(D)** and by densitometric analysis **(E)**. **(F)** Immunohistochemical staining for CIDEA, FASN and ACC in liver slices from wild-type (WT) or AKT mice administered with vehicle or HLSP. Original magnification: ×100; scale bar: 100 μm. β-actin confirms loading in the immunoblotting and qPCR assays. Three triplicate experiments were independently performed. Mean ± SD; ^#^ *P* < 0.05, ^##^ *P* < 0.01 and ^###^ *P* < 0.001 versus the WT group and *P < 0.05, **P < 0.01, and ***P < 0.001 versus the AKT group.

Following this, PPI analysis was undertaken to ascertain the dominance of certain DEGs. The analysis indicated that the node sizes for CIDEA, CIDEC, FASN, PPARG, AKT1, and ACC were significantly greater compared to other DEGs (Figure 5A), suggesting that these particular DEGs play a critical role in the protein network. Following this, key DEGs were selected to build a PPI network related to lipid metabolism using the STRING database, with the objective of identifying which DEGs are instrumental in regulating lipid metabolism (Figure 5B). ACC, FASN, PPARG, CIDEC, and especially CIDEA are all involved in the onset of hepatic steatosis and lipid droplet fusion in MASLD. Given that CIDEA is crucial for enhancing hepatic steatosis and has the ability to modulate the expression of essential genes involved in *de novo* fatty acid synthesis, namely, FASN and ACC, we delved deeper into how HLSP regulates AKT-driven lipid metabolism via CIDEA.

We used RT-PCR to examine the effect of HLSP on the mRNA expression of CIDEA, FASN, and ACC, and the results were consistent with the transcriptomics data; the mRNAs of CIDEA, FASN, and ACC were significantly overexpressed in the AKT group, and the expression was all significantly downregulated after the administration of HLSP treatment (Figure 5C). We obtained the same results at the translational level (Figures 5D, E). IHC assay further confirmed the results (Figure 5F). Overall, HLSP could attenuate hepatic steatosis by regulating the expression of key CIDEA and *de novo* fatty acid synthesis genes.

### 3.6 HLSP restrains CIDEA-mediated LD fusion and *de novo* fatty acid synthesis *in vitro*

**FIGURE 6:**
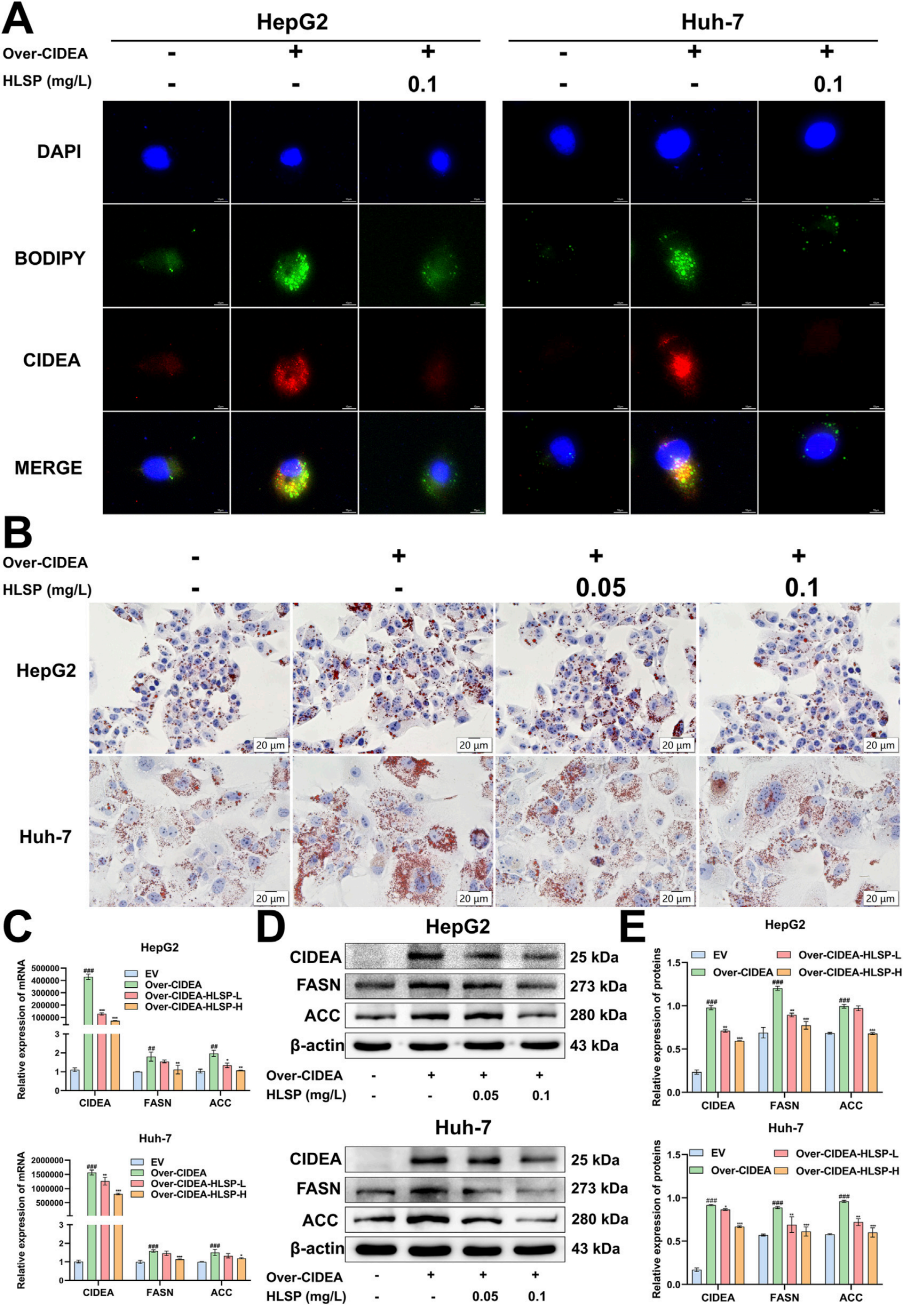
HLSP inhibits the CIDEA/FASN/ACC pathway *in vitro*. **(A)** HepG2 and Huh-7 cells were transfected with the CIDEA plasmid to overexpress CIDEA and treated with HLSP for 24 h, and then the expression of CIDEA and lipid droplets were detected. **(B)** Representative oil red O-staining images of treated HepG2 and Huh-7 cells as shown in **(A)**. **(C)** Relative mRNA expression levels of lipid metabolism genes (CIDEA, FASN, and ACC) in HepG2 and Huh-7 cells treated as indicated in **(A)**. **(D, E)** Protein expression levels of lipid metabolism genes (CIDEA, FASN, and ACC) in HepG2 and Huh-7 cells treated as indicated in **(A)**. The results are shown in the following table. Mean ± SD; ^##^ *P* < 0.01 and ^###^ *P* < 0.001 versus the WT group and *P < 0.05, **P < 0.01, and ***P < 0.001 versus the AKT group.

Finally, we investigated whether HLSP regulates adipogenesis through CIDEA. We transfected the CIDEA plasmid in HepG2 and Huh-7 hepatocellular carcinoma cells, respectively, and both observed significantly high expression of CIDEA at the protein level (Supplementary Figure S2). CCK-8 assay was performed to determine the appropriate concentration of HLSP for *in vitro* experiments (0.05–0.1 mg/L) (Supplementary Figure S3). It has been demonstrated in the literature that CIDEA promotes LDs fusion. Immunofluorescence staining was performed to determine whether HLSP can regulate LDs generation via CIDEA. The results showed that there were more and larger LDs in the cells of the Over-CIDEA group compared to the EV group, whereas CIDEA expression was reduced and LDs were reduced after the administration of HLSP intervention (Figure 6A). Meanwhile, consistent results were also obtained in ORO staining experiments (Figure 6B).

We conducted a comprehensive study at the transcriptional and translational levels to investigate whether HLSP inhibits *de novo* fatty acid synthesis through CIDEA-mediated expression of FASN and ACC. The increased expression of the CIDEA plasmid in cells demonstrated a notable rise in both mRNA and protein levels of FASN and ACC. Conversely, following the HLSP intervention, the expression levels of CIDEA, FASN, and ACC exhibited a marked decline (Figures 6C–E). Collectively, these findings indicate that HLSP impedes LD fusion and *de novo* fatty acid synthesis *in vitro* by reducing the expression of CIDEA.

## 4 Discussion

MASLD is swiftly evolving into one of the most frequent causes of chronic liver disease globally and has emerged as a primary cause of liver-associated morbidity and mortality (Mayén et al., 2024). The onset of MASLD involves hepatic lipid accumulation, and substantial hepatic fat buildup is a risk factor for the progression of the disease (Byrne and Targher, 2015). The main approaches to animal modeling of MASLD include dietary induction, pharmacological induction, and genetic engineering induction (Lau et al., 2017). However, modeling cycles are generally long and may overlook the important role of accelerated endogenous *de novo* fatty acid synthesis triggered by aberrant activation of lipid metabolism genes in the progression of MASLD. The method of using hydrodynamic injection to prepare liver disease models by directional stable transfection in the liver of living mice has the advantages of relatively short modeling time, high modeling efficiency and low mortality rate, and has been widely used in liver disease research (Calvisi et al., 2011; Wang et al., 2015). Overexpression of AKT model can effectively mimic the pathogenesis of human fatty liver, especially in lipid metabolism disorders. Here, we established a MASLD model with hepatic lipogenesis using hydrodynamic injection of AKT to investigate the protective effect of HLSP on MASLD.

In this study, we used the UPLC-Q-TOF-MS/MS technique to analyze the active ingredients in HLSP. Next, we successfully established a mouse model of MASLD by overexpressing AKT, specifically in the mouse liver. We administered HLSP to AKT mice and found that HLSP reduced liver weight and liver weight ratio in AKT mice. Elevated levels of TG and TC are indications of severe liver damage in patients with MASLD (Elshazly et al., 2015). ALT and AST are commonly used to assess whether hepatocytes are damaged or not (Valizadeh et al., 2011), and can be used as biomarkers to diagnose the progression of MASLD (Zelber-Sagi et al., 2022). Our experimental findings indicate that the HLSP intervention in AKT mice led to a decrease in TC and TG levels, as well as a reduction in ALT and AST levels. Additionally, through H&E and ORO staining, we observed that HLSP decreased lipid buildup in AKT mice. These findings suggest that HLSP mitigates hepatic steatosis induced by AKT in mice.

We performed transcriptomic analyses of liver samples to unravel the mechanism of HLSP’s action in treating MASLD further. CIDEA was identified as a key differential gene for HLSP in treating MASLD, and this is the first time that we have noted changes in CIDEA in AKT-driven mice.

Numerous studies have demonstrated that CIDEA is crucial for fat regulation. CIDEA is upregulated in steatotic livers (Matsusue et al., 2008; Hall et al., 2010; Zhou et al., 2012), and knockdown of CIDEA markedly decreases lipid accumulation in the livers of obese subjects (Xu et al., 2016). Knockdown of CIDEA in human adipocytes also significantly increased lipolysis. Additionally, higher levels of CIDEA have been noted in the livers of animals on a high-fat diet, as well as in ob/ob mice and lipodystrophic (fld) mice (Hall et al., 2010; Zhou et al., 2012). Interestingly, our findings indicated a marked increase in both mRNA and protein levels of CIDEA following AKT overexpression and the onset of hepatic steatosis. Notably, CIDEA has been shown in the literature to act as a key player in *de novo* fatty acid synthesis. Inhibition of CIDEA expression in mice resulted in varying decreases in FASN and ACC levels (Zhou et al., 2012). Furthermore, existing research has shown that silencing CIDEA in bovine mammary epithelial cells notably reduced FASN and ACC expression, whereas enhancing CIDEA levels led to an upsurge in FASN and ACC expression (Cheng et al., 2022).

Increased *de novo* fatty acid synthesis promoted the development of hepatic steatosis (Parlati et al., 2021). ACC is a lipogenic enzyme that has a strong effect on hepatic fat storage (Paul et al., 2022). FASN plays a crucial role in the final stage of *de novo* fatty acid synthesis and is regarded as a vital determinant of the liver’s ability to produce fatty acids through this process (Menendez and Lupu, 2007). Consequently, it is essential to carefully manage the expression of FASN and ACC, the primary enzymes involved in *de novo* fatty acid synthesis, as they are pivotal to lipid metabolism. In our research, we noted a marked increase in the mRNA and protein levels of CIDEA, FASN, and ACC in the livers of AKT mice. In contrast, HLSP reduces the expression of all three of the above mentioned, thereby ameliorating hepatic steatosis. This finding supports the theory that HLSP reduces *de novo* fatty acid synthesis, thereby slowing the advancement of AKT-driven MASLD by inhibiting CIDEA expression. Consistent with this, results from our *in vitro* model of CIDEA overexpression support the *in vivo* conclusion that HLSP attenuates hepatic lipid deposition by inhibiting CIDEA expression, leading to impaired *de novo* fatty acid synthesis.

LDs are organelles within cells that serve as reservoirs for neutral lipids, and their buildup in liver cells is a key characteristic of MASLD (Scorletti and Carr, 2022). Numerous studies have demonstrated that CIDEA plays a crucial role in enhancing the growth, merging, and lipid accumulation of LDs in liver cells, which can result in metabolic issues (Gong et al., 2009; Xu et al., 2012; Zhou et al., 2012). Hepatocytes from ob/ob mice displayed significantly elevated levels of CIDEA and demonstrated increased activity in lipid droplet fusion (Xu et al., 2016). In brown adipocytes lacking CIDEA, the size of LDs was markedly diminished (Zhou et al., 2003). In brown adipocytes lacking CIDEA, the size of LDs was markedly diminished (Puri et al., 2008; Zhou et al., 2012; Wu et al., 2014). Our findings from the *in vitro* model further indicated that CIDEA overexpression in hepatocellular carcinoma cells led to a notable increase in LD size, whereas the application of HLSP resulted in a significant reduction in LD dimensions.

CIDEA is a key gene regulating AKT-induced hepatic steatosis. HLSP alleviated hepatic lipoatrophy by reducing LD fusion and inhibiting *de novo* fatty acid synthesis. Similarly, we achieved comparable outcomes by overexpressing CIDEA in HepG2 and Huh-7 hepatocellular carcinoma cells.

HLSP is an adjunctive therapeutic agent for diseases such as chronic hepatitis and primary hepatocellular carcinoma. Our study is the first to investigate the therapeutic role of HLSP in MASLD. HLSP ameliorates hepatic steatosis by inhibiting CIDEA-mediated *de novo* fatty acid synthesis and LD formation. Our findings offer fresh viewpoints on how HLSP could play a role in intervening during the initial phases of MASLD progression, potentially serving as a therapeutic delay. This research sheds light on the influence of HLSP on CIDEA-mediated adipogenesis and LD formation and offers new ideas for treating MASLD with HLSP. According to our study, HLSP contains fifteen characteristic components, which may be the main medicinal substances of HLSP. Regarding the pharmacodynamic mechanisms of each component, additional research is required to uncover the potential pathways by which HLSP may treat MASLD.

## Data Availability

The data presented in the study are deposited in the OMIX repository, accession number OMIX008743.

## Abbreviations

HLSP, Hu-lu-su-pian; ACC, acetyl-CoA carboxylase; AKT, v-Akt murine thymoma viral oncogene homolog; ALT, alanine transaminase; AST, aspartate transaminase; FASN, fatty acid synthase; H&E, hematoxylin–eosin; IHC, immunohistochemistry; MASLD, metabolic dysfunction-associated steatotic liver disease; ORO, oil red O; RT-qPCR, quantitative real-time polymerase chain reaction; TC, total cholesterol; TG, triglyceride; DEGs, differentially expressed genes; MASH, metabolic dysfunction-associated steatohepatitis; FFA, free fatty acids; CIDEs, cell death-inducing DNA break factor-alpha-like effectors; CCK-8, cell counting kit-8; PCA, principal component analysis; SB, Sleeping Beauty transposase; GO, gene ontology; KEGG, Kyoto Encyclopedia of Genes and Genomes; UPLC-Q-TOF-MS/MS, ultra-high-performance liquid chromatography coupled with quadrupole time-of-flight tandem mass spectrometry.
