# Supplementary material for: Hu-lu-su-pian ameliorates hepatic steatosis by regulating CIDEA expression in AKT-driven MASLD mice
Source: Front Pharmacol. 2025 Jan 31;15:1503247. doi: 10.3389/fphar.2024.1503247 (PMC11825746; doi:10.3389/fphar.2024.1503247)
Supplement: Supplementary file 1 [file DataSheet6.docx]

**RNASeq Method**

**Sample collection and preparation**

**1.RNAquantification and qualification**

RNA degradation and contamination was monitored on 1% agarose gels.

RNA purity was checked using the NanoPhotometer® spectrophotometer (IMPLEN, CA, USA).

RNA concentration was measured using Qubit® RNA Assay Kit in Qubit®2.0 Flurometer (Life Technologies, CA, USA).

RNA integrity was assessed using the RNA Nano 6000 Assay Kit of the Bioanalyzer 2100 system (Agilent Technologies, CA, USA)

**2.Library preparation for Transcriptome sequencing**

Atotal amount of 1 μg RNA per sample was used as input material for the RNA sample preparations.

Sequencing libraries were generated using NEBNext®UltraTM RNA Library Prep Kit for Illumina®(NEB, USA) following manufacturer’s recommendations and index codes were added to attribute sequences to each sample. Briefly, mRNA was purified from total RNA using poly-T oligo attached magnetic beads. Fragmentation was carried out using divalent cations under elevated temperature in NEBNext First Strand Synthesis Reaction Buffer(5X). First strand cDNA was synthesized using random hexamer primer and M-MuLV Reverse Transcriptase (RNase H-). Second strand cDNA synthesis was subsequently performed using DNA Polymerase I and RNase H. Remaining overhangs were converted into blunt ends via exonuclease/polymerase activities. After adenylation of 3’ends of DNA fragments, NEBNext Adaptor with hairpin loop structure were ligated to prepare for hybridization. In order to select cDNA fragments of preferentially 250~300 bp in length, the library ragments were purified with AMPure XP system (Beckman Coulter, Beverly, USA). Then 3 μl USER Enzyme (NEB, USA) was used with size-selected, adaptor-ligated cDNA at 37°C for 15 min followed by 5 min at 95°C before PCR. Then PCR was performed with Phusion High-Fidelity DNA polymerase, Universal PCR primers and Index (X) Primer. Atlast, PCR products were purified (AMPure XP system) and library quality was assessed on the Agilent Bioanalyzer 2100 system.

**3.Clustering and sequencing**

The clustering of the index-coded samples was performed on a cBot Cluster Generation System using TruSeq PE Cluster Kit v3-cBot-HS (Illumia) according to the manufacturer’s instructions. After cluster generation, the library preparations were sequenced on an Illumina platform and 150 bp paired-end reads were generated.

**Data Analysis**

**1.Data quality control**

Use fastp v 0.19.3 to filter the original data, mainly to remove reads with adapters; when the N content in any sequencing reads exceeds 10% of the base number of the reads, remove the paired reads; when any sequencing reads When the number of low-quality (Q<=20) bases contained in reads exceeds 50% of the bases of the reads, this paired reads will be removed. All subsequent analyses are based on clean reads.

**2.Reads mapping to the reference genome**

Download the reference genome and its annotation files from the designated website, use HISAT v2.1.0 to construct the index, and compare clean reads to the reference genome.

**3.Quantification of gene expression levels**

Use featureCounts v1.6.2 to calculate the gene alignment and FPKM. FPKM is currently the most commonly used method to estimate gene expression levels.

**4.Difference analysis**

DESeq2 v1.22.1/edgeR v3.24.3 was used to analyze the differential expression between the two groups, and the *P* value was corrected using the Benjamini & Hochberg method. The corrected *P* value and |log_2_foldchange| are used as the threshold for significant difference expression.

**5.Differential gene enrichment analysis**

The enrichment analysis is performed based on the hypergeometric test. For KEGG, the hypergeometric distribution test is performed with the unit of pathway; for GO, it is performed based on the GO term.

**6.Differential AS analysis**

Use rMATS v3.1.0 to analyze variable splicing events, including five alternative splicing events: SE, RI, MXE, A5SS, andA3SS.

**7.SNPanalysis**

Use GATK v4.1.9.0 to analyze the variant sites, and use annovar to annotate the variant sites.

**8.fusion gene analysis**

STAR-Fusion v1.5.0 software was used to detect fusion transcripts using the fusion output of STAR v2.6.1d alignments.

**9.Differential gene protein interaction analysis**

The protein interaction analysis of differentially expressed genes is based on the STRING database of known and predicted protein-protein interactions, building networks based on known interactions of species.
