# Supplementary material for: Hu-lu-su-pian ameliorates hepatic steatosis by regulating CIDEA expression in AKT-driven MASLD mice
Source: Front Pharmacol. 2025 Jan 31;15:1503247. doi: 10.3389/fphar.2024.1503247 (PMC11825746; doi:10.3389/fphar.2024.1503247)

**Supplementary Table S1.** List of the primary antibodies used for Western blot analysis (WB)

| **Protein** | **Antibody (and catalog number)** | **Application** |
| --- | --- | --- |
| FASN | Rabbit monoclonal (3180) | WB, IHC † |
| ACC | Rabbit monoclonal (3676) | WB, IHC † |
| CIDEA | Rabbit Polyclonal (13170-1-AP) | WB, IHC # |
| β-actin | Rabbit monoclonal (4970) | WB † |

† Provided by Cell Signaling Technology Inc. (Danvers, MA).

# Provided by Proteintech (Wuhan, China).

**Supplementary Table S2.** Sequences of primers used in quantitative real-time PCR (qPCR)

| **Gene** | **Sequences (5′ to 3′)** | **Source** |
| --- | --- | --- |
| FASN | Forward: TGGTCTTTCTGTGCTTGGATT  Reverse: GGAGTCTTGGCAGGGTGGA | Human |
| ACC | Forward: ATGTCTGGCTTGCACCTAGTA  Reverse: CCCCAAAGCGAGTAACAAATTCT | Human |
| CIDEA | Forward: CTCATCAGGCCCCTGACATT  Reverse: CGGCATCCACTTCTGTCCTT | Human |
| β-actin | Forward: GTCCACCGCAAATGCTTCTA  Reverse: TGCTGTCACCTTCACCGTTC | Human |
| FASN | Forward: ACCTGCCCAATCTCTATAGTGTC  Reverse: CCACACAGCCTCGTAAGGGA | Mouse |
| ACC | Forward: CCAGCAGAATTTGTTACTCG  Reverse: TTCACTGCTGCAATACCAT | Mouse |
| CIDEA | Forward: CCTACGACATCCGATGCACA  Reverse: TATCCACGCAGTTCCCACAC | Mouse |
| β-actin | Forward: CGTTGACATCCGTAAAGACCTC  Reverse: TAGGAGCCAGGGCAGTAATCT | Mouse |

**Supplementary Table S3.** The key reagent information

| **Reagent** | **Catalog number** | **Manufacturer** |
| --- | --- | --- |
| Protease inhibitors | CW2200S | Jiangsu Cowin Biotech Co., Ltd. |
| Phosphatase inhibitors | CW2383S | Jiangsu Cowin Biotech Co., Ltd. |
| CCK-8 | CK04 | Dojindo Laboratories |
| Triglyceride assay kit | A110-1-1 | Nanjing Jiancheng Bioengineering Institute |
| Total cholesterol assay kit | A111-1-1 | Nanjing Jiancheng Bioengineering Institute |
| Alanine aminotransferase  Assay Kit | C009-2-1 | Nanjing Jiancheng Bioengineering Institute |
| Aspartate aminotransferase  Assay Kit | C010-2-1 | Nanjing Jiancheng Bioengineering Institute |
| ECL ultrasensitive  chemiluminescent solution | SQ201 | Shanghai Epizyme Biomedical Technology Co., Ltd. |
| Oil Red O Stain Kit,  For Cultured Cells | G1262 | Beijing Solarbio Science & Technology Co., Ltd. |
| BODIPY 493/503 | MX5403-5MG | Shanghai Maokang Biotechnology Co., Ltd. |
| Universal Tissue Fixative | G1101-500ML | Wuhan Servicebio Technology Co., Ltd. |
| PBS | G4202-500ML | Wuhan Servicebio Technology Co., Ltd. |
| CIDEA plasmid | RC205790 | OriGene |
| MegaTran 2.0 plasmid  transfection reagent | TT210002 | OriGene |
| Fetal Bovine Serum | 10099-141 | GiBco |
| Dulbecco’s modified  eagle medium | SH30022.01 | HyClone |
| Trypsin 0.25% Solution | SH30042.01 | HyClone |

**Supplementary Figure S1.** The ESI and structure of Cucurbitacins in HLSP


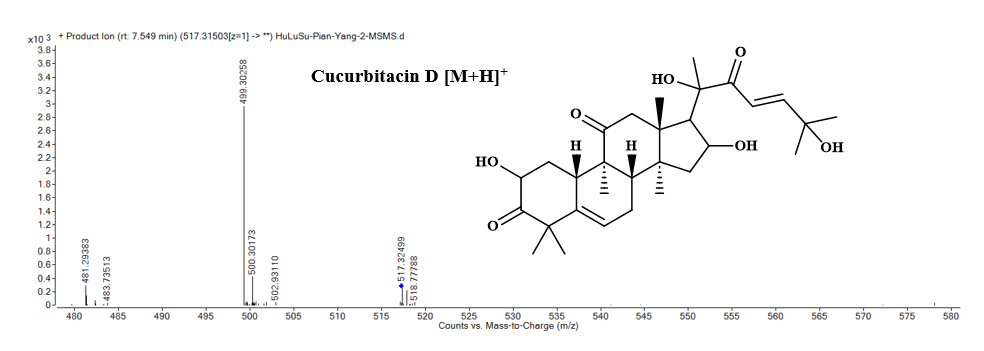


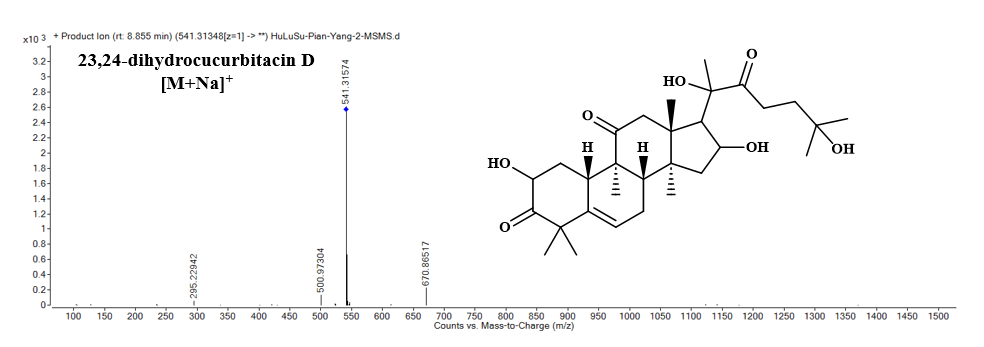


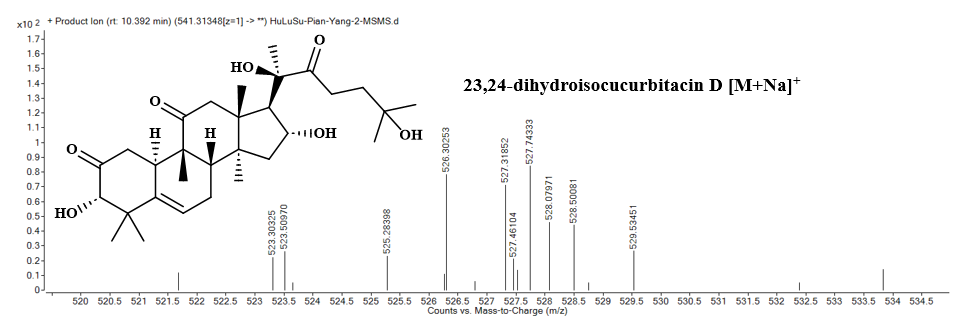


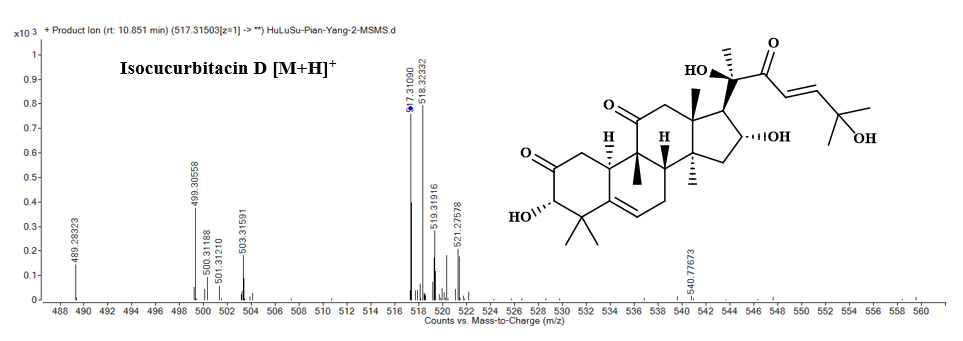


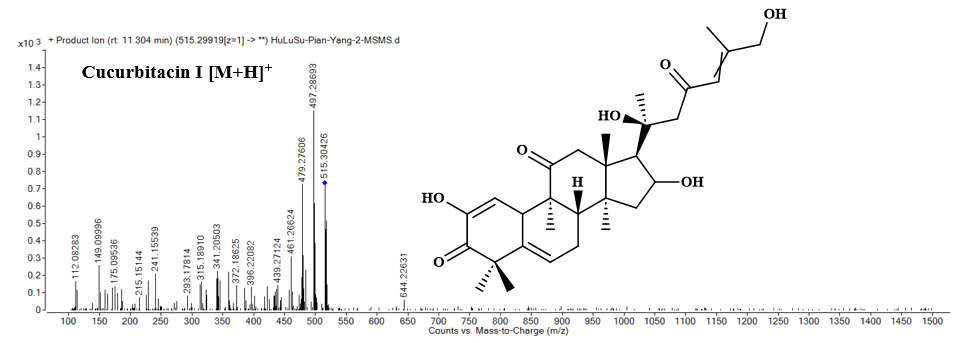


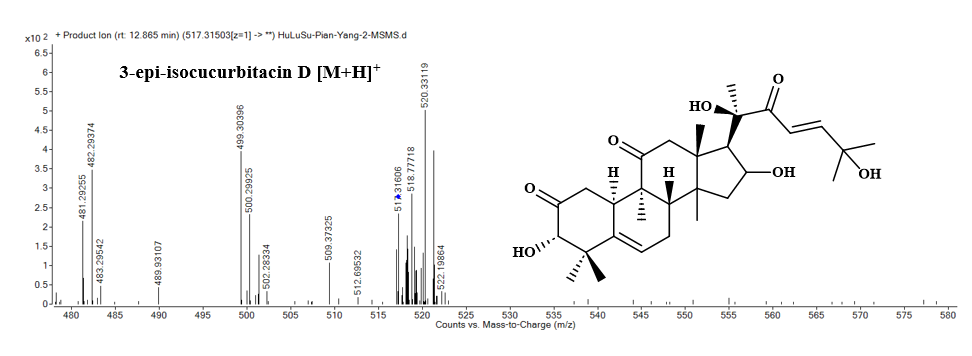


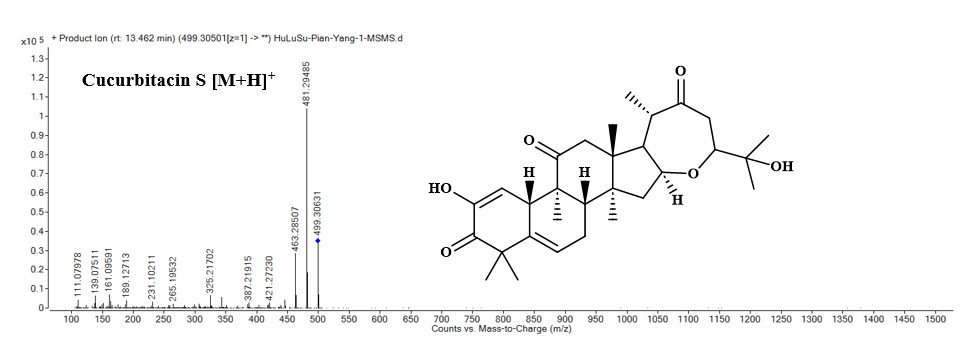


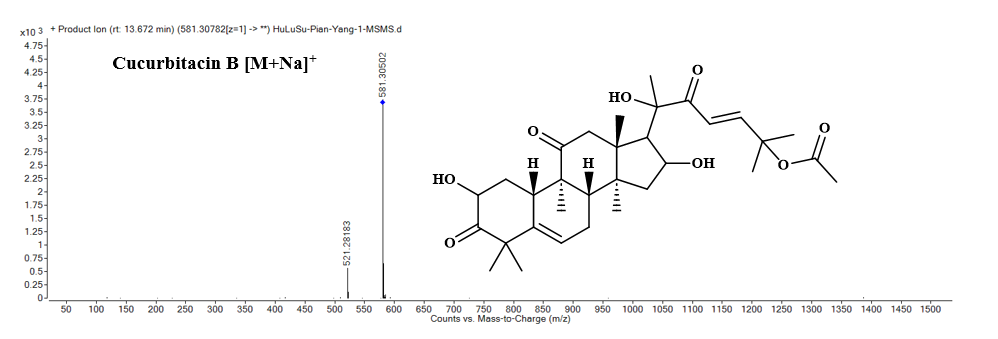


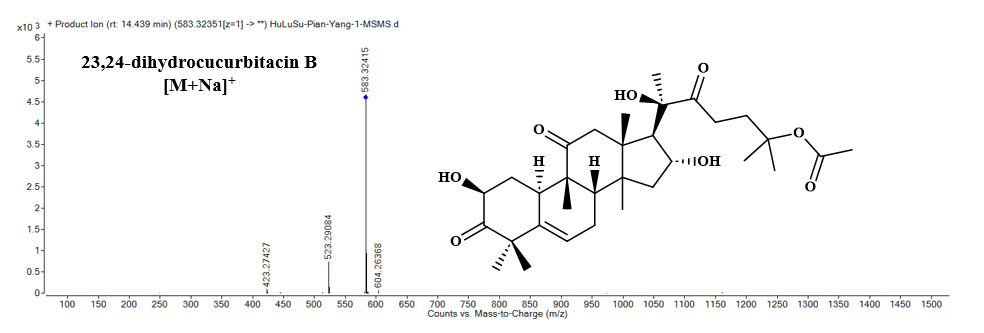


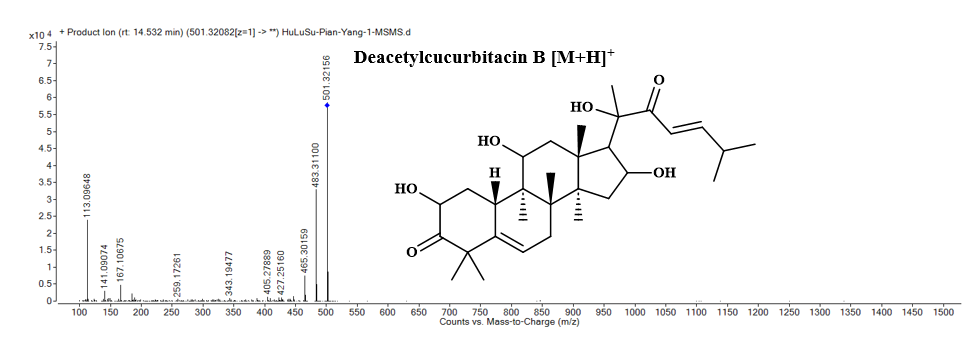


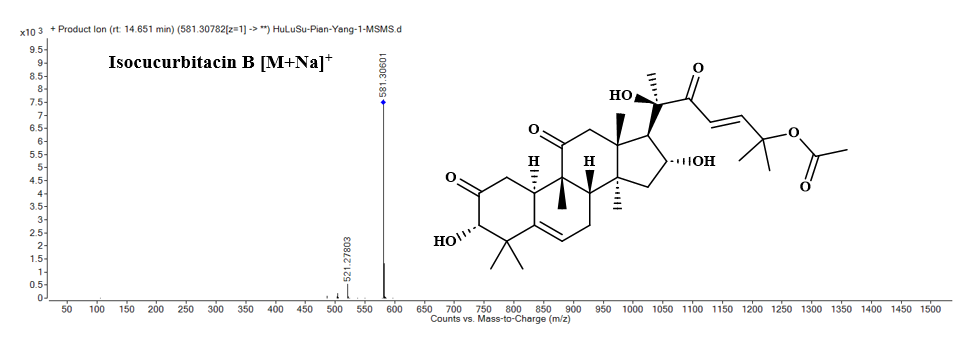


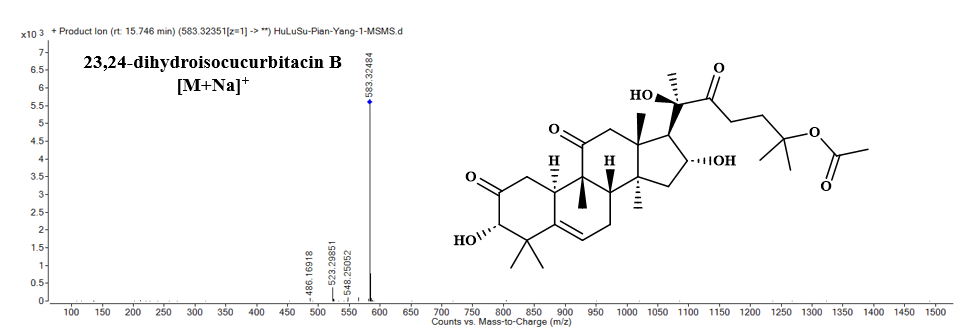


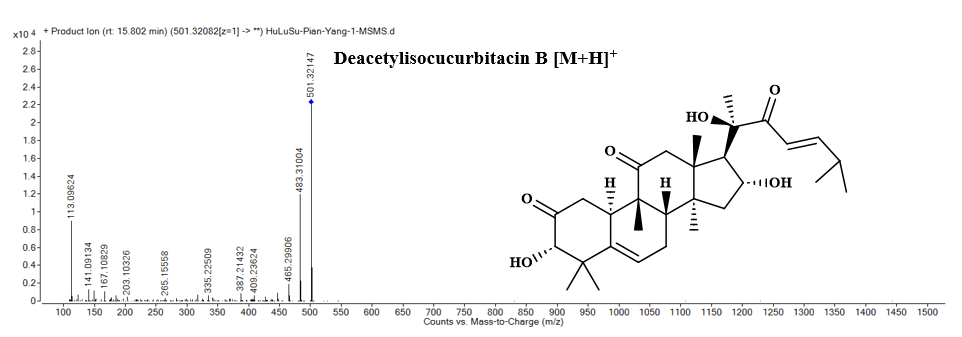


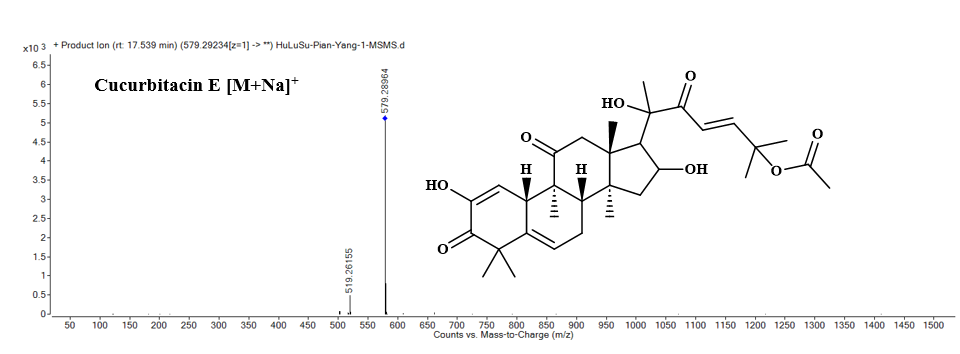


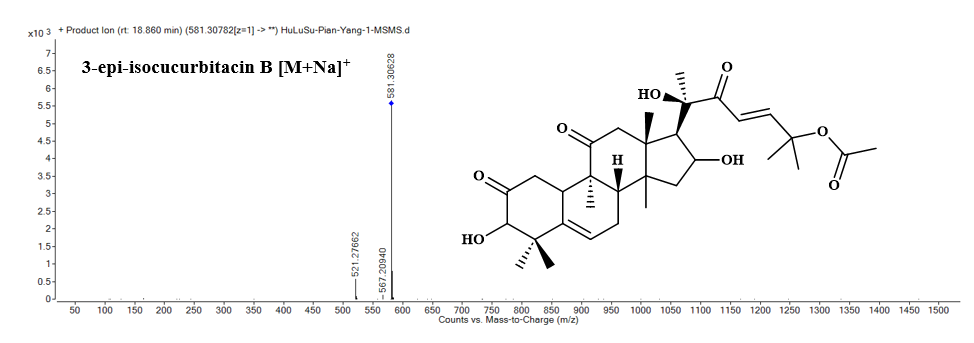


**Supplementary Figure S2.** The protein expression profile of CIDEA plasmid transfected into HepG2 and Huh-7 cells

**
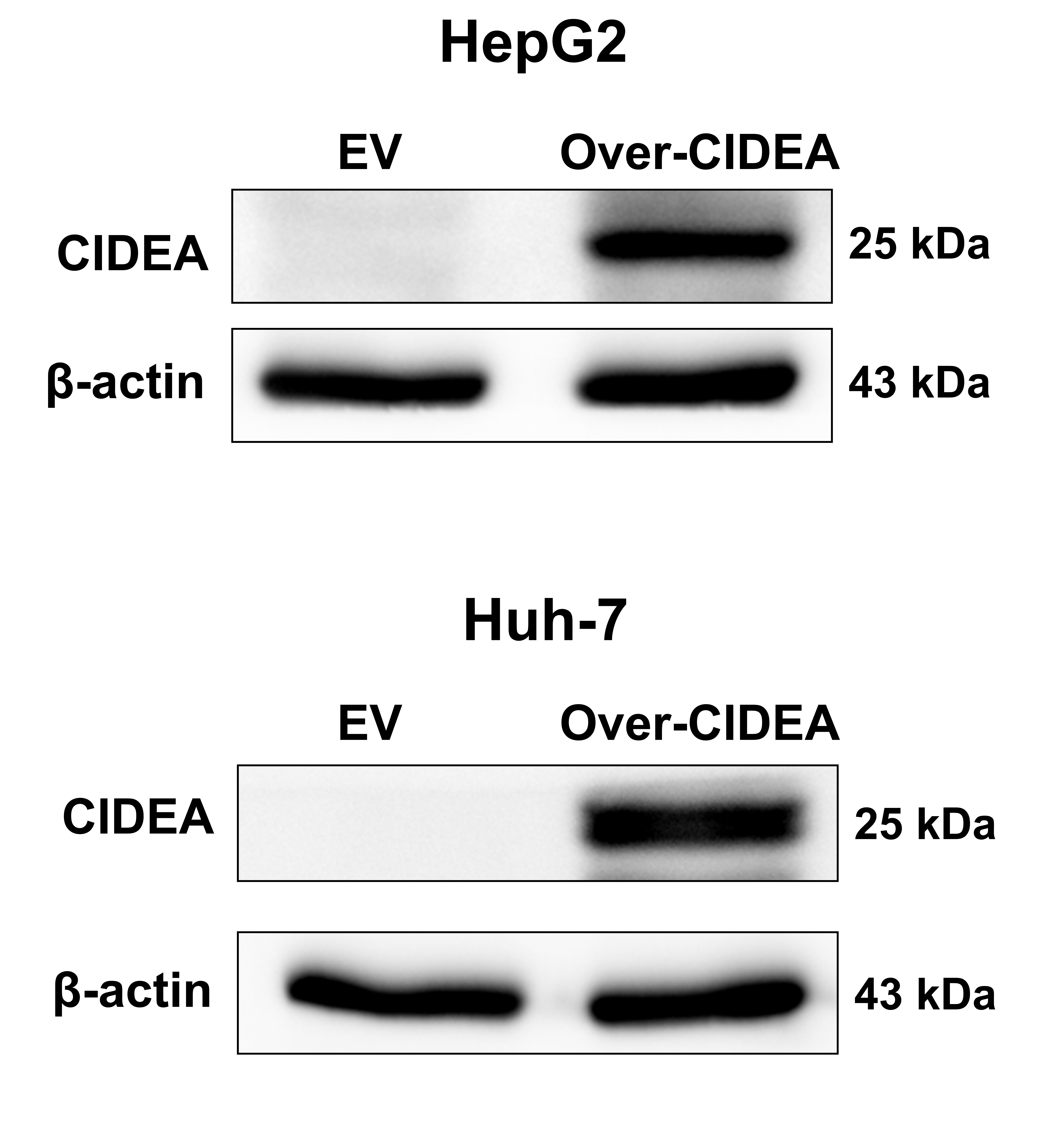

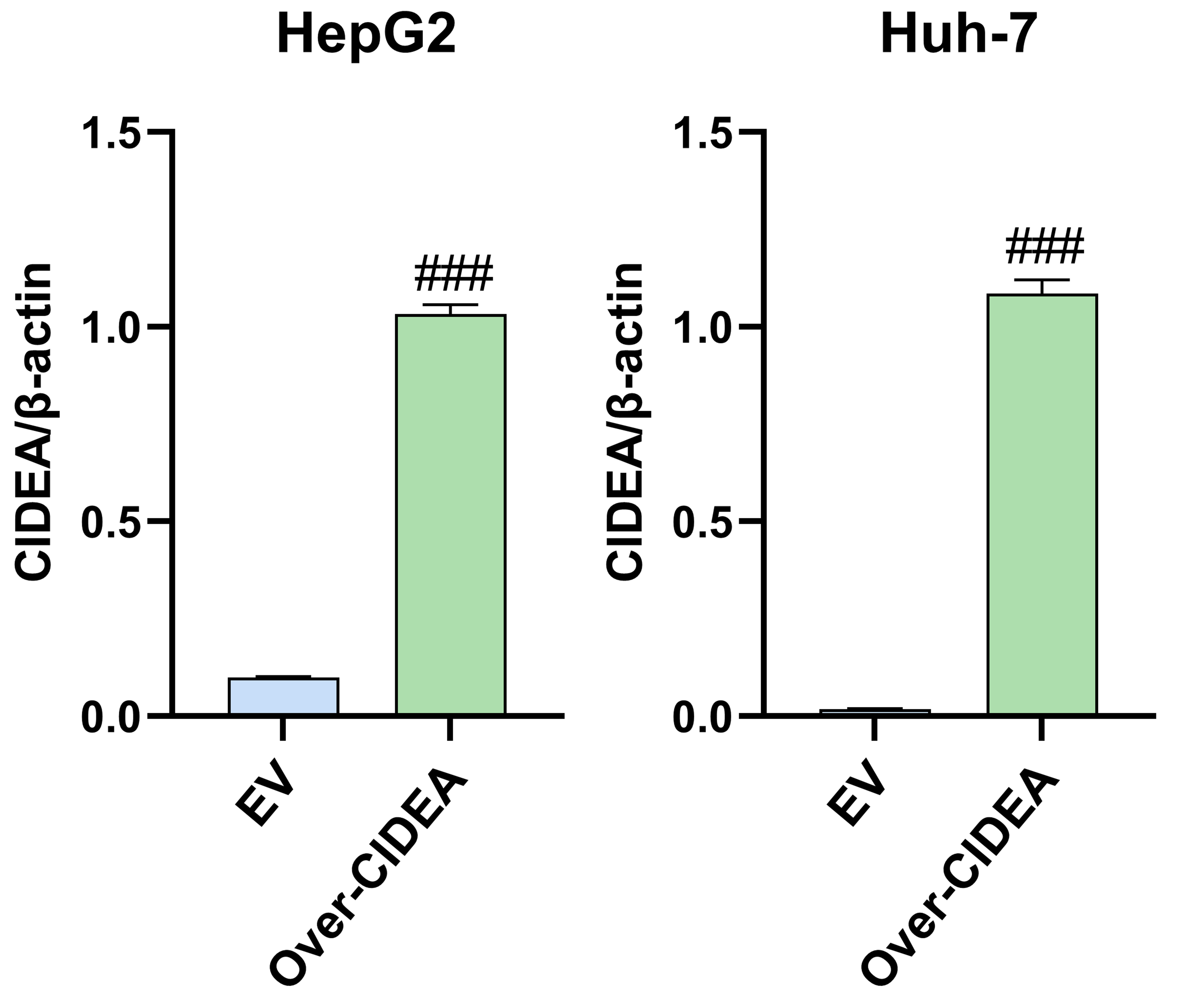
**

**Supplementary Figure S3.** Concentrations of HLSP administered in *in vitro* experiments


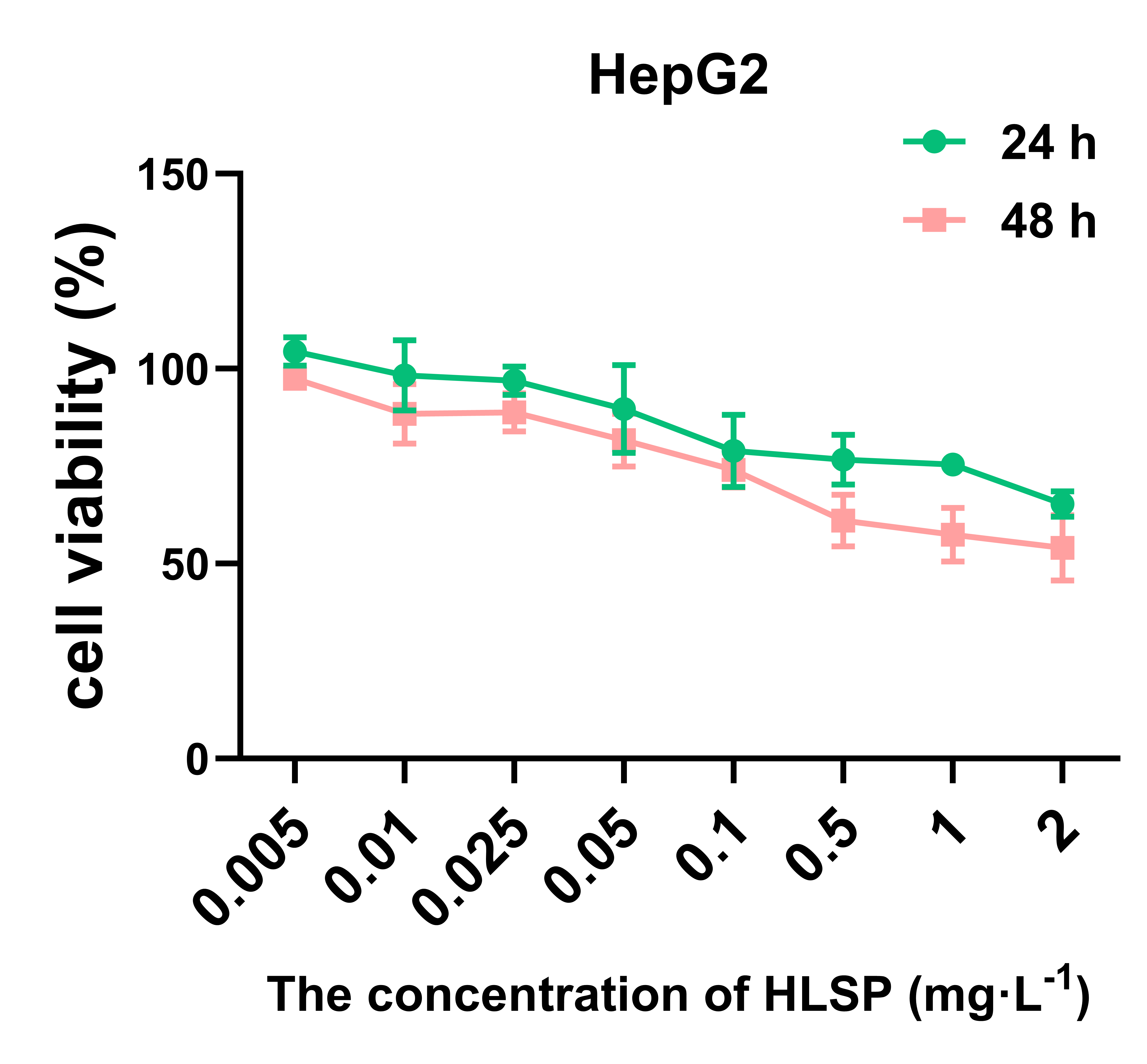

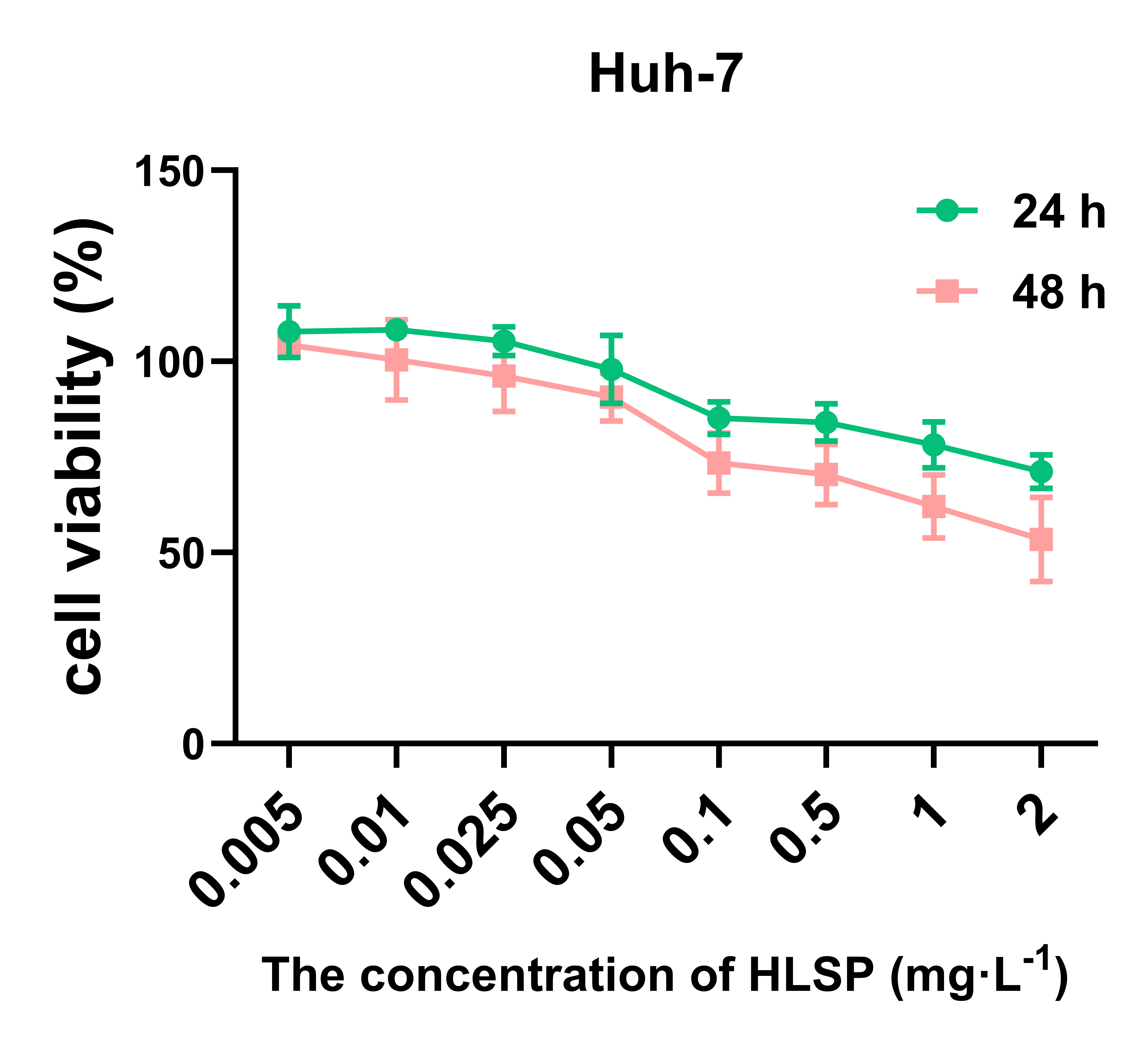

Supplement: Supplementary file 5 [file DataSheet1.docx]
